# Donor-Specific Blood Transfusion in Lung Transplantation

**DOI:** 10.3389/ti.2024.12822

**Published:** 2024-10-30

**Authors:** Xin Jin, Jacques Pirenne, Robin Vos, Charlotte Hooft, Janne Kaes, Jan Van Slambrouck, Phéline Kortleven, Christelle Vandervelde, Hanne Beeckmans, Pieterjan Kerckhof, Marianne S. Carlon, Dirk Van Raemdonck, Mark R. Looney, Bart M. Vanaudenaerde, Laurens J. Ceulemans

**Affiliations:** ^1^ Department of Chronic Diseases and Metabolism (CHROMETA), Laboratory of Respiratory Diseases and Thoracic Surgery (BREATHE), KU Leuven, Leuven, Belgium; ^2^ Department of Thoracic Surgery, University Hospitals Leuven, Leuven, Belgium; ^3^ Department of Microbiology, Immunology and Transplantation, Transplantation Research Group, Lab of Abdominal Transplantation, KU Leuven, Leuven, Belgium; ^4^ Department of Abdominal Transplantation, University Hospitals Leuven, Leuven, Belgium; ^5^ Department of Respiratory Diseases, University Hospitals Leuven, Leuven, Belgium; ^6^ Department of Oncology, Laboratory of Angiogenesis and Vascular Metabolism (VIB-KU Leuven), KU Leuven, Leuven, Belgium; ^7^ Department of Medicine, University of California, San Francisco (UCSF), San Francisco, CA, United States; ^8^ Department of Laboratory Medicine, University of California, San Francisco (UCSF), San Francisco, CA, United States

**Keywords:** immunosuppression, lung transplantation, tolerance, transplant immunology, donor-specific blood transfusion

## Abstract

Lung transplantation is still hindered by a high rate of chronic rejection necessitating profound immunosuppression with its associated complications. Donor-specific blood transfusion is a pre-transplant strategy aimed at improving graft acceptance. In contrast with standard stored blood or donor-specific regulatory T cells transfusions, this approach utilizes fresh whole blood from the donor prior to allograft transplantation, encompassing all cell types and plasma. The precise mechanisms underlying donor-specific blood transfusion-induced tolerance remain incompletely understood. Associations with regulatory/helper T cells, modulation of mononuclear phagocytic cells or microchimerism have been suggested. While numerous (pre-)clinical studies have explored its application in solid organ transplants like liver, kidney, and intestine, limited attention has been given to the setting of lung transplantation. This comprehensive review summarizes existing knowledge on the mechanisms and outcomes of donor-specific blood transfusion in solid organ transplants both in preclinical and clinical settings. We also address the potential benefits and risks associated with donor-specific blood transfusion in the field of lung transplantation, offering insights into future research directions.

## Introduction

An important milestone was reached in 2022 with 70,000 adult lung transplantations (LTx) being performed over the past three decades according to the International Society for Heart and Lung Transplantation. LTx is a last resort for patients with end-stage pulmonary disease but the outcome remains limited with an internationally reported 5-year survival rate of 59% [1, 2]. After LTx, the recipient’s immune system identifies the allograft as “non-self,” activating a robust alloimmune response due to major histocompatibility complex (MHC) incompatibility between donor and recipient. Antigen-presenting cells (APC) trigger the maturation of upstream naïve immune cells into effector T or B cells. This intricate immunological process is also characterized the production of cytokines such as interleukin-2 (IL-2) and interferon-gamma (INF-γ) and mediated by regulatory T cells (Treg) and/or B cells (Breg) population. Effective immunosuppression after LTx is crucial to prevent rejection and subsequent alloimmune injury to the lung [3–5].

However, chronic and profound immunosuppressive therapy induces drug toxicity (renal and cardiovascular toxicity, neurotoxicity, etc.) and increases susceptibility to infections and malignancies [6, 7]. Despite heavy immunosuppression, a higher rate of chronic lung allograft dysfunction (CLAD) is observed, compared to other transplantations such as liver and kidney. The future of LTx hinges on the prospect of widening the patient’s therapeutic window improving graft acceptance without resorting to profound immunosuppression.

Before the era of modern calcineurin inhibitor-based immunosuppression, donor-specific blood transfusion (DSBT) has been used to facilitate graft acceptance [8, 9]. It involves the infusion of donor whole blood to recipients prior to transplantation, with the potential to improve graft acceptance or even induce donor-specific tolerance. In contrast to standard transfusion of blood products like red blood cells, platelets, or plasma, DSBT involves the use of whole blood directly obtained from the donor, containing all blood cell types and plasma proteins.

**FIGURE 1:**
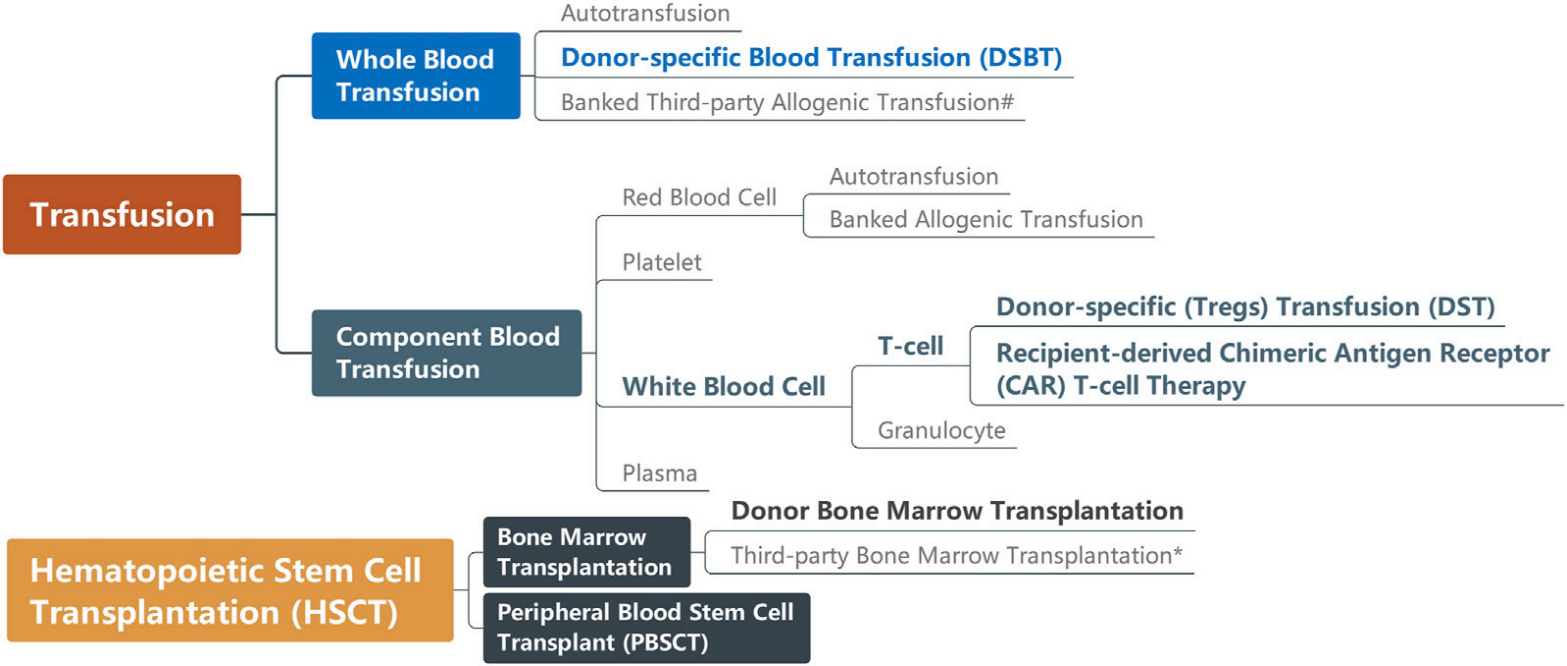
Pan-transfusion techniques relevant to solid organ transplantation. Techniques (in bold) are reported as capable of improving graft acceptance. ^#^Before transfusion, third-party whole blood usually is irradiated for leukoreduction; *Bone marrow transplantation causing graft-versus-host disease (GvHD) is the indication for some solid organ transplantation.

The definition of DSBT changed over time, leading to confusion about the concept in the literature. Initially, the research referred to this therapy was called donor-specific transfusion, abbreviated as DST [10]. However, advancements in blood apheresis techniques have narrowed the DST definition down to the transfusion of specific subpopulations of donor leukocytes (especially Tregs), resembling chimeric antigen receptor (CAR) T-cell immunotherapy in oncology, which has also been recently reported that the recipient-derived CAR-T cells targeting patients’ B cells are capable of improving allograft acceptance after kidney transplantation [11–13]. Moreover, donor hematopoietic stem cell transplantation is also reported to permit solid organ allograft survival with preconditioning such as thymic irradiation, sublethal whole body irradiation and T cell depletion but without immunosuppression in several animal experiments and clinical trials [14–17]. Consequently, the crucial aspect of the original DSBT concept now lies in the transfusion of whole blood [DSBT(WB)]. Pan-transfusion techniques relevant to solid organ transplantation are summarized in Figure 1.

## The Mechanism of DSBT-Induced Tolerance in Transplantation

**FIGURE 2:**
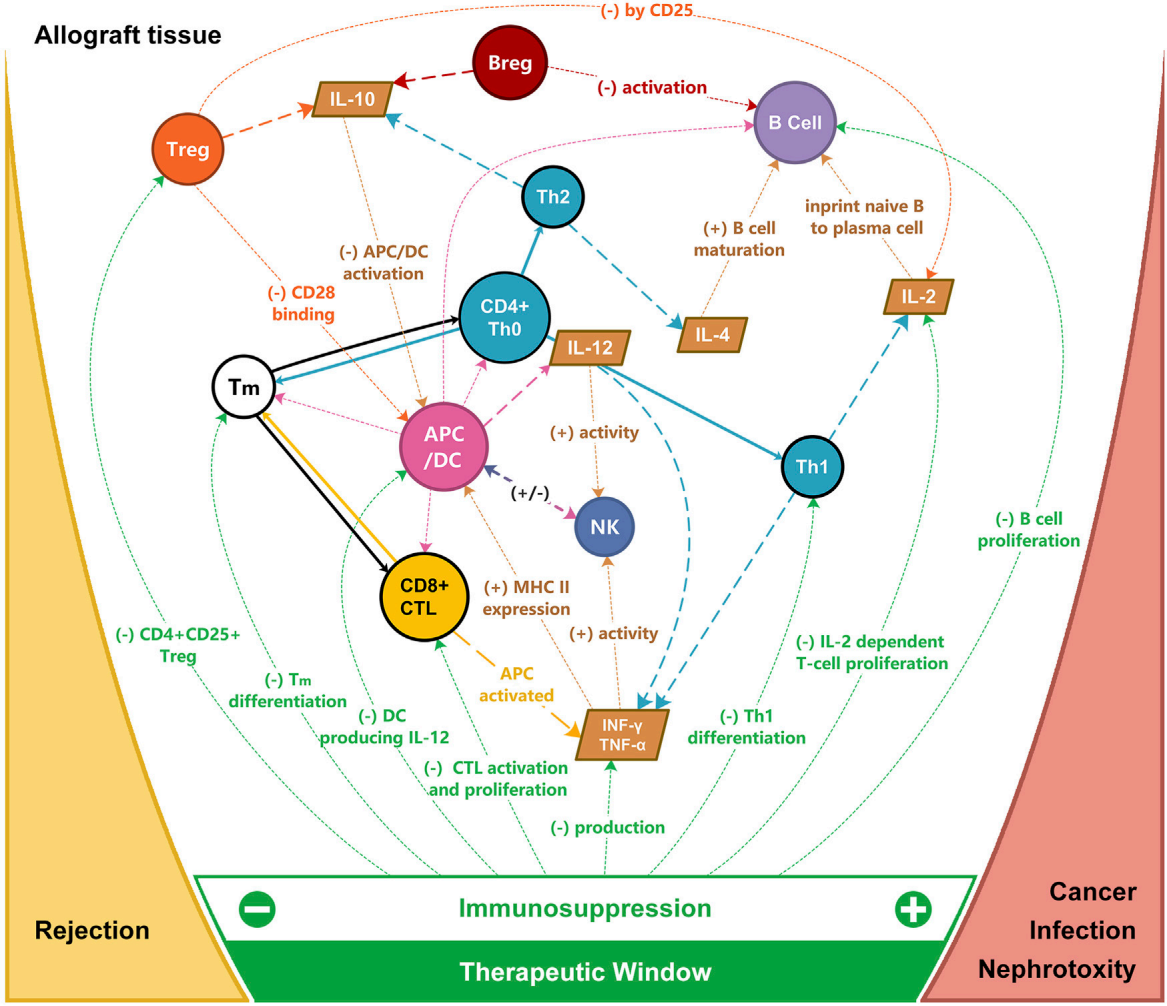
Targets of immunosuppression for allograft rejection and the therapeutic window for immunosuppression. APC, antigen-presenting cells; Breg, regulatory B cell; CTL, cytotoxic T cell; DC, dendritic cell; IL, interleukin; INF, interferon; NK, nature killer cell; TNF, tumor necrosis factor; Th, T helper cell; Tm, memory T cell; Treg, regulatory T cell.

In current medical practice, the alloimmune response is non-specifically blocked to maintain graft acceptance by immunosuppressive drugs such as calcineurin inhibitors, antimetabolites, and anti-interleukin monoclonal antibodies (Figure 2) [18]. For example, corticosteroids inhibit pro-inflammatory gene expression and promote the expression of anti-inflammatory cytokines and transcription mediators [19, 20]. Posttransplant survival was hampered by their non-specific action and their severe metabolic adverse effects. To address this challenge, various “tolerogenic” approaches were explored. Tolerance refers to a state of acceptance without immunosuppression, while prope tolerance is reached with a limited amount of immunosuppression. For instance, the use of donor spleen cells, epidermal cells, skin extract, and whole blood were utilized in skin transplant experiments in an attempt to promote graft acceptance [21, 22].

In 1963, Halasz et al. noted that improving dog skin graft survival was achieved by subcutaneous injection of 2 mL donor blood 10 and 5 days prior to transplantation. This approach demonstrated superior outcomes compared to transplantation without prior blood injection or with blood from a third-party donor (26 days vs. 10 days and 16 days, respectively) [23]. Subsequently, in 1964 in a canine allogeneic kidney transplant model, they demonstrated that pre-treatment with subcutaneous injection of 2 mL donor blood 10 and 5 days before transplantation extended graft survival from 8 to 29 days. Of note, transfusion immediately after transplantation followed by repeated transfusion every 5th day prolonged survival albeit more modestly to 16 days [10]. Fabre and Morris later replicated these findings in a rat renal transplant model [(DA × Lewis)F1 → DA/Lewis] in 1972. Intravenous injection of 0.5 mL donor strain blood was given 1 or 7 days before transplantation or twice weekly for periods of 4 or more weeks. Longest survival was observed in the 7-day group [24]. Subsequent validation of irradiated DSBT in a rat pancreatic islet transplant model [Lewis (RT1^I^) → ACI (RT1^a^)] and non-irradiated DSBT with anti-CD28 antibody in a liver transplant model [DA (RT1^a^) → Lewis(RT1^I^)] also confirmed the DSBT potential to improve graft acceptance and recipient survival [25, 26].

In 1980, Salvatierra et al. documented the first human application of DSBT in living donor renal transplantation with a one-haplotype match. A volume of 200cc of fresh (within 24 h) whole blood or equivalent packed cells (considering the regional blood bank preferences in subunit amount of blood and logistics of transfer or mailing blood from geographically distant donors) was administered three times at a two-week interval before living-donor transplantation. Immunosuppression was initiated 2 days before transplantation. No hyperacute or accelerated rejection was observed in 23 DSBT-treated patients who had lower 3-month rejection (44% vs. 82%), and higher 1-year graft survival (94% vs. 56%), compared to untreated patients with high mixed lymphocyte culture index. A total of 239 cases were monitored during 4 years. The graft and patient survival rate of recipients with 0 and 1 haplotype treated with DSBT were comparable to HLA-identical recipients without DSBT (graft survival: 82% vs. 84%; 4-year patient survival: 95% vs. 93%) [8, 9]. Our experience of the Leuven Immunomodulatory Protocol for human intestinal transplantation consists of the peritransplant administration of 400–600 mL of DSBT, along with a modified low immunosuppressive regimen and a series of maneuvers (ischemia and infection-free donor, selective bowel decontamination and glutamine administration, synchronizing donor and recipient surgery for a short ischemic time, etc.) aimed at promoting a low-inflammatory/pro-regulatory environment. No chronic rejection occurred in 13 treated intestinal transplant recipients with a 5-year graft/patient survival of 92% compared to a 5-year graft survival of 58% and patient survival of 61% [27, 28].

**FIGURE 3:**
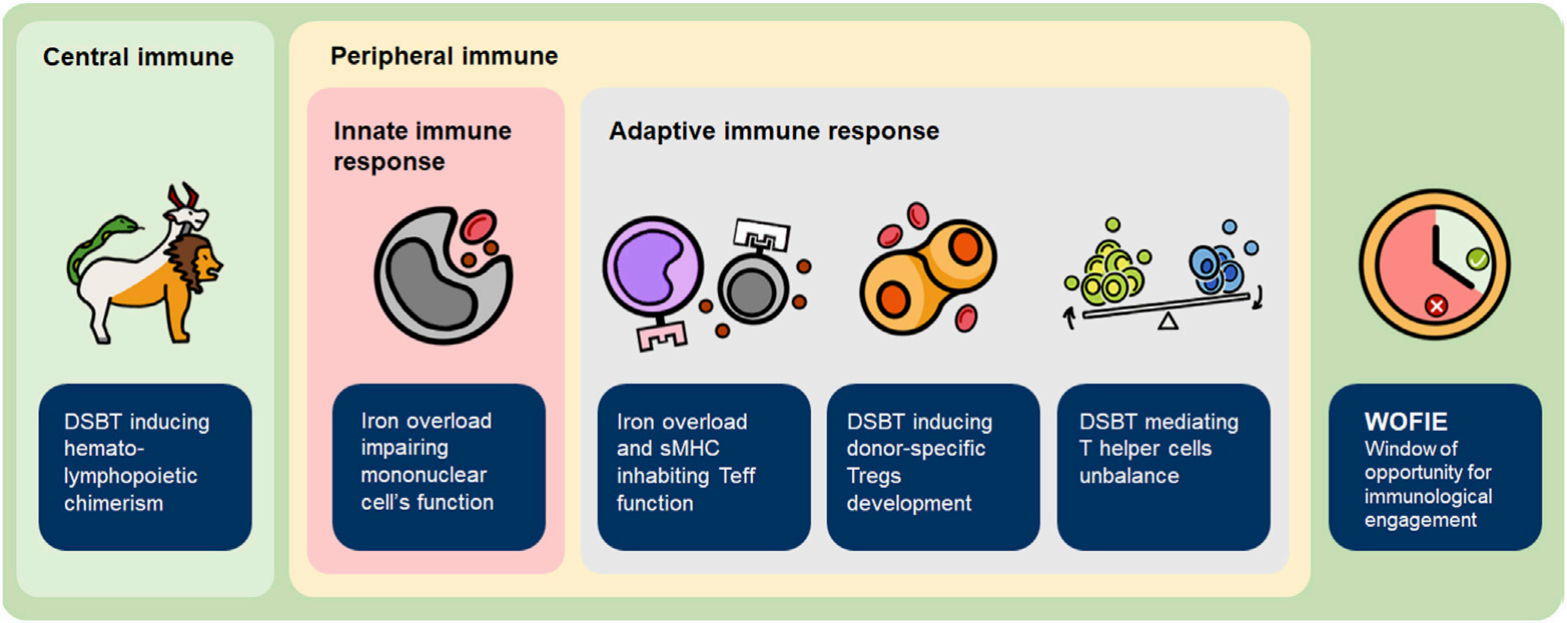
Mechanism of how DSBT induces (donor-specific) tolerance. sMHC, soluble major histocompatibility complex molecules; Teff, effector T cell; Treg, regulatory T cell.

The potential benefits of DSBT have been demonstrated but the mechanisms by which DSBT operate remain unclear. Various hypotheses have been formulated (Figure 3):

### DSBT With Transplantation Induces Hematolymphopoietic Chimerism

Chimerism refers to the stable persistence of a group of cells in another genetically distinct individual. In microchimerism (MC) the circulating cell population is below 5%. MC can be observed after non-leukoreduced and leukoreduced blood product transfusion and in transplant recipients, twins, and pregnant women [29, 30]. The lifespan of peripheral blood cells varies from hours to around 100 days [31]. Transfused leukocytes are expected to be completely cleared by alloimmune recognition and/or natural cell senescence. However, Lee et al. observed a transient proliferation of donor white blood cells in canine and human recipients circulation 3–5 days after unmanipulated packed RBC transfusion [32, 33]. Transfusion-associated microchimerism (TA-MC) was observed in 45% of severe trauma surgery patients and sometimes lasted for years [33, 34]. TA-MC may initially result from the proliferation of passenger leukocytes and, in the long term, from the differentiation of donor peripheral blood stem cells (PBSC), which can be present in the peripheral circulation at any time [35].

Like in trauma surgery, transplantation recipients may also suffer from peri-operative fluid loss, prolonged involuntary thermoregulation under anesthesia and/or extracorporeal life support, ischemia-reperfusion injury, and infection risks due to exposure to the environment (e.g., pathogens in recipient bronchus could contaminate the chest cavity during LTx). Their immune system is over-stressed, which creates a favourable environment for TA-MC to exist and continue to regulate recipient’s immune system long-term after transplantation.

In a mouse allogenic femur transplant model, Bingaman et al. found that hematolymphopoietic chimerism (due to bone marrow transplantation) could lead to long-term donor-specific hyporesponsiveness [36]. In 1999, Spitzer et al. conducted a histocompatibility leukocyte antigen (HLA)-matched bone marrow and kidney transplant on a preoperatively induced female patient with multiple myeloma and end-stage renal disease. The low dose of cyclosporine monotherapy was completely withdrawn on Day 73. Renal function remained stable, with no evidence of acute or chronic rejection, and the patient survived over 5 years after transplantation [15, 16].

Starzl et al. proposed a two-way paradigm to explain how MC can induce tolerance. The outcome of transplantation is influenced by both host-versus-graft (HvG) and graft-versus-host (GvH) immune reactions, regulated by the migration and localization of the respective immunogenic leukocytes. If the donor antigen could primarily bypass or secondarily avoid collection by recipient lymphoid tissue, where the passenger leukocytes preferentially migrate to, the immune response could not be induced and the recipient could remain ignorant of the graft existence. This process is mediated by multiple cytokine and receptor pathways. For example, the persistence of both immune reactions could trigger mutual clonal exhaustion-deletion through FasL and tumor necrosis factor (TNF) pathways, which would be crucial for tolerance induction [37–40].

### DSBT(WB) Impairs Mononuclear Phagocytic Cells and Effector T Cells Function

Following blood transfusion, a large amount of iron released from damaged red blood cells and present in plasma is phagocytized by monocytes. The iron homeostasis relevant intra-graft gene expression can predict tolerance in liver transplantation. A higher serum level of hepcidin and ferritin and increased hepatocyte iron deposition were found in operationally tolerant liver transplant recipients [41]. The extracellular iron levels and the balance among ferritin generation and secretion and the primary form of ferrous iron storage play a crucial role in monocyte function [42, 43].

Excessive cellular iron level can impair transcription factor regulation, such as reducing activation of nuclear factor interleukin 6 (NF-IL6) and hypoxia-inducible factor-1 α (HIF-1α) and inhibiting phosphorylation of signal transducer and activator of transcription 1 (STAT1) [44]. NF-IL6 plays a central role in cytokine and iron-mediated regulation of nitric oxide synthase (NOS) expression. Reduced NF-IL6 can downregulate the expression of inflammatory cytokines (such as IL-1 and TNF) and granulocyte colony-stimulating factor (G-CSF) in mature macrophages [45, 46]. The absence of HIF-1α results in ATP level decreasing in macrophages further reducing phagocytosis and migration. HIF-1α deletion can cause a reduction of inflammatory cytokines such as IL-1β, IL-6, IL-10, TNF, and IFN-γ in macrophage and/or dendritic cells (DCs). HIF-1α is also essential for pro-inflammatory M1-type macrophage polarization and maturation of DCs [47–49]. STAT1 can regulate the number and phenotype of macrophages [50]. The STAT1 deficiency can abolish STAT1-dependent cellular response to both INF-α and -γ resulting in immunodeficiency [51, 52].

Iron overload can also directly affect the expansion and function of effector T cells. In patients with hereditary hemochromatosis, where iron overload is a prominent feature, the proliferative capacity, numbers, and activity of cytotoxic T cells (CD8^+^CD28^+^) were decreased, while the number of CD8^+^CD28^−^ T cells was increased [53–55]. Abundant CD8^+^CD28^−^ T cell numbers were associated with better graft function and reduced rejection by inhibiting antigen-presenting cell (APC) allo-stimulatory capacity in liver transplant patients [56]. Consequently, iron overload impairs phagocytosis and antigen presentation leading to decreased activation of effector T cells [57, 58].

Additionally, soluble MHC class I and II molecules (sMHC-I and sMHC-II) are distinctive components in DSBT(WB) compared to regularly stored red blood cell transfusions. sMHC molecule carries donor tolerogenic peptides that can bind to recipient T cells through T cell receptors (TCR). sMHC-TCR binding competing with recognition by APCs results in receptor blockade and apoptosis of recipient T cells due to the absence of co-stimulatory molecules [59–61]. Calne raised a concept of “the liver effect” to describe the immunosuppressive effect of liver transplantation on the other allograft [62]. Graeb et al. further showed in a rat [ACI(RT1^a^) → Lewis (RT1^I^] model that donor liver-produced sMHC could suppress immune response in recipients and protect heart allograft from rejection [63]. The balance between sMHC-I and II has also been reported to be linked to immune homeostasis [64, 65].

Taken altogether, DSBT(WB) has the potential to block both the antigen presentation of mononuclear phagocytic cells and the activation of effector T cells. Transplantation in such an environment could therefore facilitate graft acceptance [66].

### DSBT Induces the Development of Donor-Specific Tregs After Transplantation

Tregs account for 5%–10% of the T cell population in peripheral blood and contribute to immune homeostasis by regulating innate and adaptive immune responses [67]. Particularly CD4^+^ Tregs expressing forkhead box protein 3 (Foxp3) have been shown to regulate alloimmune response after transplantation. In both animal studies and clinical research, it was found that Tregs interact with other effector T cells through inhibitory cytokines, cytotoxicity, and direct contact [68–71]. Compared to deleukocyted blood product, donor-Tregs could be transfused into recipients through DSBT(WB) and survive due to TA-MC, further mediating immune response after transplantation. Furthermore, it has been observed in a rat model [ACI (RT1.A^a^B^b^) → Lewis (RT1.A^l^B^l^)] that DSBT can also induce active expansion of CD4^+^ T cells and donor-specific Tregs in the DSBT recipient’s spleen by indirect allorecognition via residents DCs [72].

In the skin transplant model, CBA/Ca (H-2^k^) mice received weekly intravenous transfusions of 0.25 mL whole blood from donor C57BL/10 (H-2^b^) for five cycles. CD4^+^CD25^+^ T cells from the mesenteric lymph nodes and spleens of transfused mice, collected 1 week after the final transfusion, were co-transferred with naïve CD45RB^hi^ cells (effector cells) into CBA-Rag deficient recipient mice 1 day before skin transplantation. This protocol induced long-term tolerance, with 100% skin allograft survival after 100 days without immunosuppression [73]. Similar outcomes were observed in rat heart and intestinal transplants [RA (RT1^p^-RT1A^c^:B/D^c^) → PVG (RT1^c^-RT1A^u^:B/D^l^)], where DSBT induced the development of CD4^+^CD45RC^−^ Tregs in recipients from post-transplant Day 5. These Tregs were highly effective in transferring donor-specific tolerance, as confirmed by adoptive cell transfer experiments. In addition to DSBT, the generation of Tregs requires the presence of graft, thymus, and spleen. These Tregs can be found in secondary lymphoid tissues and in the graft itself, suggesting a local protective effect [73–75].

Human transplant studies also support the role of Tregs in graft acceptance. In Leuven Immunomodulatory Protocol-treated intestinal transplantation recipients, a high level (1.8%) of circulating CD4^+^CD45RA^−^Foxp3^hi^ memory Tregs was detected in the graft, correlating with long-term reduction of rejection [27]. Furthermore, the Foxp3^hi^ Tregs subset was associated with improved outcomes graft and patient survival after kidney transplantation in a cohort of donor-specific hypo-responders [76–78].

### DSBT Can Mediate the T helper Cells Unbalance Against Acute Rejection

Secretion of pro-inflammatory cytokines (IL-2, IL-12, TNF-α, INF-γ, etc.) by type 1 CD4^+^ T helper cells (Th1) promotes differentiation of cytotoxic CD8^+^ T cells, natural killer (NK) cells and macrophages which are associated with acute rejection. Conversely, the function of type 2 CD4^+^ T helper cells (Th2) is more complex and depends on cellular targets, timing, and Th2 cytokine-dependent Tregs. Th2 produce cytokines such as IL-4, IL-6, IL-10 and IL-13 with both pro- and anti-inflammatory functions and hereby facilitate Tregs, mediate macrophage polarization to M2 phenotype, and trigger B cells humoral immune response. Th2 are considered to be more linked to chronic rejection [79–84]. The balance of Th1/Th2 is implicated in the level of immune response post-transplantation.

In the rat model of DSBT [RA (RT1^p^-RT1A^c^:B/D^c^) → PVG (RT1^c^-RT1A^u^:B/D^l^)], the Th1/Th2 cytokine profile differed, and a Th2 bias was observed after heart transplantation. On post-transplant day (POD) 5, INF-γ and IL-10 levels in allograft-rejecting rats peaked significantly higher than in DSBT-tolerized rats, whose levels did not peak until POD 9 and POD 30, respectively. IL-4 levels in DSBT-tolerized rats continued to rise until POD 30, while in allograft-rejecting rats, it peaked on POD 5 and was considerably lower than in DSBT-tolerized rats [85]. The mechanism by which DSBT modulates this Th1/Th2 balance remains unclear. A potential explanation is Tregs expansion after/by DSBT [86]. sMHC-I in DSBT may also regulate Th1/Th2 cytokine expression by decreasing IL-2 and granulocyte-macrophage colony-stimulating factor (GM-CSF) while increasing IL-4 and IL-15 [59].

### DSBT May Stimulate the Regulatory Immunologic Mechanisms During “the Window of Opportunity for Immunological Engagement (WOFIE)” When the Immunosuppression is Postponed Initially for 72–96 h

The WOFIE theory is based on the two-way paradigm hypothesis of Calne and Starzl [38, 87–89]. Tolerance, like rejection, is an active immune response that relies on the balance between graft-versus-host (GvH) and host-versus-graft (HvG) reactions. This balance, crucial for graft survival, is influenced by the graft’s immunogenicity, the recipient’s immune system, and donor immune cells, resulting in graft rejection, tolerance, or GvHD. Despite the potential for HLA sensitization, DSBT does not provoke a strong or sustained immune response. Instead, DSBT creates a window of opportunity for immune engagement by promoting a balanced interaction between graft-versus-host (GvH) and host-versus-graft (HvG) responses.

**FIGURE 4:**
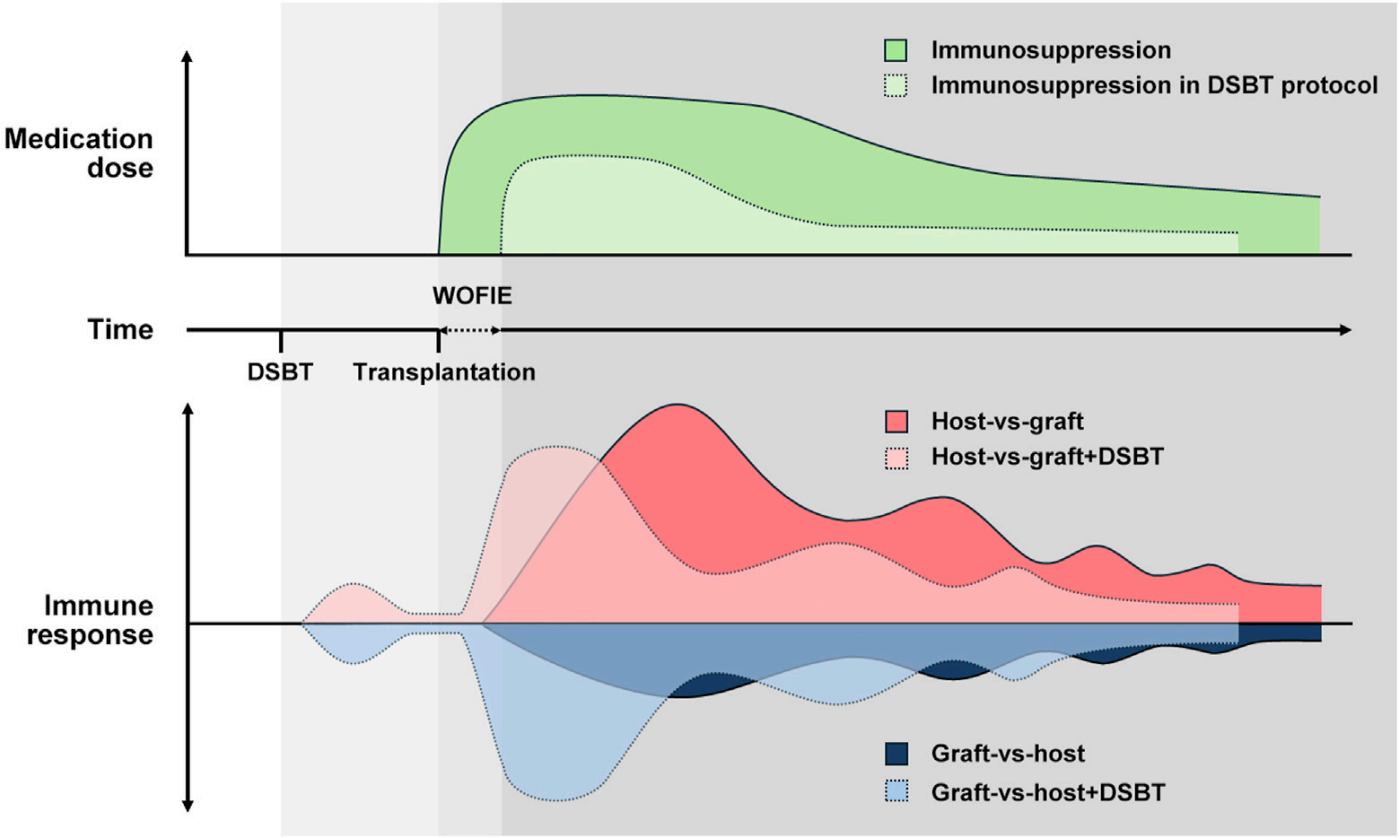
How DSBT benefits from WOFIE and stimulates the regulatory immunologic mechanism according to the two-way paradigm hypothesis by Calne and Starzl. Despite the potential for HLA sensitization, DSBT does not provoke a strong or sustained immune response but creates a window of opportunity for immune engagement by promoting a balanced interaction between GvH and HvG responses. Non-specific excessive immunosuppression given before and immediately after the transplant can block this naturally occurring well-balanced tolerogenic response. On the longer-term lower levels of maintenance immunosuppression can prevent an overactive HvG response, thereby maintaining the balance and tolerogenic interaction.

Early after the allograft is exposed to the recipient’s immune system, the GvH response is higher and HvG is lower in recipients who have received DSBT compared to those who have not. However, this increased GvH response remains balanced with decreased HvG, facilitating the development of immunological tolerance and potentially improving graft survival. Non-specific excessive immunosuppression given before and immediately after the transplant can block this naturally occurring well-balanced tolerogenic response. On the longer-term lower levels of maintenance immunosuppression can prevent an overactive HvG response, thereby maintaining the balance and tolerogenic interaction (Figure 4).

Calne et al. first introduced the concept of WOFIE in porcine renal transplant in 1994, administering irradiated leukocytes from the donor spleen to the recipient 6 h after transplantation, and creating a cyclosporine-free window of 48 h [88]. In 1998 they replaced leukocytes with DSBT on the day of transplantation and prolonged the cyclosporine-free window to 96 h in rhesus monkeys kidney transplantation model [87]. In both models, this limited immunosuppression strategy was proved to be effective in improving graft acceptance. In rat intestinal transplantation [RA (RT1^p^-RT1A^c^:B/D^c^) → PVG (RT1^c^-RT1A^u^:B/D^l^)], Pirenne et al. demonstrated that DSBT-induced tolerance could be disrupted by high doses of methylprednisolone [90]. They further observed in rat heart transplantation [RA (RT1^p^-RT1A^c^:B/D^c^) → PVG (RT1^c^-RT1A^u^:B/D^l^)] that a low level of cyclosporine (10 mg/kg) given peri-transplant could lead to tolerance whereas a high level (50 mg/kg) compromised graft acceptance and recipient survival, by blocking the development of T regs. Additionally, administering 10 mg/kg cyclosporine on POD 0–4 failed to induce tolerance, but proved effective when given on POD 5–9 [91].

## Need to Explore the Potential Protective Effect of DSBT in LTx

In contrast to animal and clinical research in kidney, liver, heart and intestinal transplantation, the role of DSBT in LTx has not been explored. However, DSBT holds an important potential in addressing the shortcomings of postoperative immunosuppression after LTx. The conventional immunosuppressive regimen following lung transplantation typically consists of induction immunosuppression (anti-thymocyte globulin or Basiliximab) and a triple-drug combination of maintenance immunosuppression comprising a calcineurin inhibitor (cyclosporine or tacrolimus), an antiproliferative agent (azathioprine, mycophenolate, sirolimus, or everolimus), and corticosteroids (methylprednisolone and prednisone) [92, 93].

The lung is highly susceptible to rejection. The lung is a lymphoid organ exposed to the outside environment, accounting in part for its immunogenicity. In addition, epithelial cells can function as antigen-presenting cells and directly activate CD4^+^ cells [94]. The immunogenicity of the lung renders LTx recipient dependent upon heavier chronic immunosuppression, compared to liver and kidney transplants, where patients may more easily transit to dual- or monotherapy with lower drug levels [95, 96]. Moreover, liver and kidney transplants can be performed with living donors. However, LTx is almost exclusively performed with organs from deceased donors, and therefore, the preoperative induction window is limited. LTx’s prognosis is significantly inferior to other organs [97]. New strategies are necessary to overcome the issue.

Compared to DST, which faces limitations such as the unpredictable selection of donor cells, extended *ex vivo* proliferation time, and an elevated risk of combining with monoclonal antibodies, DSBT has the potential to be a more applicable and practical option in the setting of LTx for reducing postoperative immunosuppression with fewer technical and ethical constraints.

To translate the experience of DSBT in other solid organ transplants and further understand its mechanism, we need to first verify the safety of DSBT in LTx and rule out three severe complications:

### Transfusion-Related Acute Lung Injury (TRALI)

TRALI is characterized by the onset of new acute lung injury within 6 hours of a blood transfusion, with no identifiable other risk factors. TRALI can occur in all kinds of blood products transfusion but most frequently in products with >60 mL of plasma [98]. The incidence of TRALI is 0.2‰, making it a leading cause of mortality associated with plasma-containing transfusions in the United States [99, 100]. Diagnosing TRALI can be challenging, particularly in distinguishing it from primary graft dysfunction (PGD) following LTx, as both complications present similar symptoms of hypoxemia and bilateral infiltrates on chest X-ray [99, 101]. Despite this similarity, TRALI is strongly linked to blood transfusions, while PGD may occur up to 72 h after LTx.

The exact mechanism of TRALI remains unclear, but it is believed to be triggered by donor leukocyte antibodies present in blood products [102]. Risk factors for TRALI include major surgery within 72 h, active infection, massive transfusion, and cytokine administration, which primes circulating hematopoietic cells before encountering antibodies, thereby increasing the risk of TRALI [99, 103–105]. Currently, there is no published data on TRALI in the DSBT animal model. In our own preclinical experience of DSBT in mice LTx, we did not observe any event of TRALI after iso- or allo-blood transfusion.

### Hyperacute and Acute Rejections Due to “Transfusion-Related Sensitization” in Recipients

Hyperacute rejection rarely occurs after LTx, clinically featuring sudden hypoxemia, widespread pulmonary infiltration, and newly developed pulmonary hypertension within hours after reperfusion [106, 107]. Acute rejection is more common in about 10% of all adult LTx recipients within 1 year posttransplant [108]. In both rejections, preformed and *de novo* donor-specific antibodies (DSA) are the risk factors [109]. It has been confirmed in a rat model [ACI (RT1.A^a^B^b^) → Lewis (RT1.A^l^B^l^)] that DSBT can induce antibody-forming cells to produce DSA in the spleen [72]. Therefore, DSBT may sensitize recipients resulting in increased risks of hyperacute and acute rejection.

However, Ueta et al. found in the rat model that the *de novo* DSA were not detectable until Day 5 and reached a peak concentration on Day 7. These DSA targeted MHC-I on donor passenger T cells and suppressed acute GvHD [72]. While it is the anti-DQ (HLA-II) DSA that is more often considered associated with antibody-mediated rejection and worse prognosis in LTx [110–112]. Pirenne et al. also reported no hyperacute or acute rejection events in their DSBT-treated intestinal transplant patients [27]. Whether DSBT-induced DSA can sensitize LTx recipients and cause hyperacute and acute rejections remains controversial and should be closely monitored.

### Transfusion-Associated Graft Versus Host Disease (TA-GvHD)

TA-GvHD is a rare fatal complication after blood transfusions. It is characterized by pancytopenia and multiple organ failure, likely triggered by the proliferation of donor T cells in the circulation. These cells not only engraft but also attack host tissues, mirroring the pattern of GvHD [113, 114]. While case reports of TA-GvHD have been observed after liver, lung, and kidney transplantation [115–117], the underlying mechanisms remain inadequately explored.

TA-GvHD is particularly problematic in immune immature recipients, such as infants, due to their inability to recognize and eliminate foreign donor cells, coupled with the presence of shared HLA antigens, which are identified as primary risk factors [118–120]. In the DSBT protocol, the fresh whole blood is not irradiated and deleukocyted, which is an effective preventive measure against TA-GvHD [121]. For transplantation patients, partial HLA matching, especially when the donor is homozygous for an HLA haplotype while the recipient is heterozygous, results in the situation that the recipient’s immune system fails to identify and clear the donor-specific leukocytes. In contrast, the donor leukocytes are activated to target the recipient tissue [122]. Transplant recipients could also be immunodeficient due to poor preoperative status and induction immunosuppression during the window of DSBT, which raises the risk of TA-GvHD. Retrospective analysis suggests that the dose of lymphocytes, approximately 10^7^ lymphocytes/kg of recipient weight, correlates with the risk of susceptible TA-GVHD cases [123]. It suggests that the volume of DSBT is not “the more, the better,” and the preoperative evaluation should be more cautious based on the HLA haplotype status.

An animal model of DSBT in LTx could provide essential insight into determining the best timing and dose of DSBT, immune cell differentiation after DSBT and the role of TA-MC in immune regulation. A previously published review has discussed the pros and cons of animal LTx models [124], and we propose that the rodent model stands out as a more suitable choice for DSBT research in LTx, considering factors such as cost, surgical complexity, and availability of analysis techniques.

## Feasibility of DSBT in LTx

Although it requires days before transplantation for DSBT’s induction and after transplantation for WOFIE to induce tolerance in animal models, It has been proven in clinical liver, kidney and intestinal transplantations that DSBT is a feasible method combined with revised immunosuppression plan to improve graft/patient survival (summarized in Table 1) [9, 27, 125–127]. New hypothermic storage equipment permits preoperative organ preservation for a longer time up to a maximum of 24 h, ensuring an adequate WOFIE, starting before transplantation and finishing at least before reperfusion or even earlier, for DSBT treatment in LTx [128, 129].

**TABLE 1:** Clinical DSBT applications in different solid transplantations.

Year	Title	Donor	Organ	DSBT		Immunosuppression		Survival
				Volume	Time	Plan	Time	
1985 [9]	A seven-year experience with donor-specific blood transfusions. Results and considerations for maximum efficacy	Living	Kidney	200 mL/time	3 sperate occasions at approximately 2 week intervals	Azathioprine, prednisone (ATGAM for steroid resistant rejection) and cyclosporine (after introducing to clinics)	Since 2 days before transplantation	Patient 4-Year: 95%
2011 [125]	Beneficial effects of donor-specific transfusion on renal allograft outcome	Living	Kidney	150 mL/time	3 sperate occasions at approximately 2 week intervals	Azathioprine, methyl-prednisolone,cyclosporine	Since 2 days before transplantation	Patient 5-Year: 92.8% 8-Year: 81.5%
2016 [27]	The Leuven Immunomodulatory Protocol Promotes T-Regulatory Cells and Substantially Prolongs Survival After First Intestinal Transplantation	Deceased	Intestine	400–600 mL	One time collected during procurement, transfused perioperatively and finished before reperfusion	Induction: basiliximab or Thymoglobulin Maintenance^#^: tacrolimus, steroids, azathioprine and Thymoglobulin (refractory rejection)	Since POD 0	Patient 5-Year: 92%
2021 [127]	The use of organ donor blood in liver transplantation	Deceased	Liver	4 Units	One time collected during procurement, transfused perioperatively when needed for critical bleeding	Not mentioned	Not mentioned	Patient 5-Year: 87% 10-Year: 80%
2021 [126]	The Impact of Intraoperative Donor Blood on Packed Red Blood Cell Transfusion During Deceased Donor Liver Transplantation: A Retrospective Cohort Study	Deceased	Liver	500–900 mL	One time collected during procurement, transfused perioperatively when needed for critical bleeding	Not mentioned	Not mentioned	Graft Long-term*: 97.1%

ATGAM, lymphocyte immune globulin; POD, postoperative day; ^#^ dose-decreased protocol; * which is defined as no retransplantation after 30 postoperative days.

Our recent published data has already proven that DSBT is feasible and safe within the mice model with the same species setting as our mice LTx model [Balb/c (H2^d^) → C57BL6/N(H2^b^)]. We observed no histological changes in mice’s lung tissue or complications including fluid overload after a single DSBT, but the ratio of circulatory lymphocytes dropped after allo-transfusion compared to iso-transfused mice [C57BL6/N(H2^b^)→ C57BL6/N(H2^b^)] [130]. The ongoing pilot study observed a sequential hematological evolution and potential of immunoregulatory modulation with different DSBT protocols in mice LTx.

In conclusion, DSBT has demonstrated improved graft outcomes following solid organ transplantation (including liver, kidney, heart, and intestine) in various animal models and clinical studies. These findings could be applicable to LTx as well. Therefore, establishing a new animal model and protocol for LTx, along with further investigation into the underlying mechanisms, is essential.
